# Understanding Digital Dementia and Cognitive Impact in the Current Era of the Internet: A Review

**DOI:** 10.7759/cureus.70029

**Published:** 2024-09-23

**Authors:** Zeeshan Ali, Jayaprakash Janarthanan, Prasanna Mohan

**Affiliations:** 1 Physiology, Krupanidhi College of Physiotherapy, Bengaluru, IND; 2 Physiotherapy, Harsha Institute of Physiotherapy, Bengaluru, IND; 3 Physiotherapy, Krupanidhi College of Physiotherapy, Bengaluru, IND

**Keywords:** adhd, cognition decline, dementia, digital wellbeing, internet

## Abstract

Dementia encompasses symptoms resulting from brain damage that impairs cognitive functions, surpassing natural aging effects. This condition affects emotional regulation, behavior, and motivation while preserving consciousness. Dr. Manfred Spitzer coined the term 'digital dementia,' highlighting the cognitive decline associated with excessive reliance on digital devices such as smartphones and Google, potentially exacerbating attention deficit hyperactivity disorder (ADHD) and memory loss. This condition mirrors terms like 'digital amnesia' and 'the Google Effect,' highlighting the brain's tendency to offload peripheral information, leading to panic and forgetfulness. Spitzer's book, *Digital Dementia,* focuses on gaming effects on children and has thus popularized the term. Teenagers are known to use electronic devices regularly, correlating with rising cognitive impairments.

The advent of the internet's fifth generation (5G) has transformed technology use, impacting mental health treatments and clinical practices globally. Digital media's influence on the developing brain encompasses motor skills, language, and cognition. Excessive digital media use in young adults correlates with lower cognitive empathy, affecting interpersonal understanding and facial recognition. Studies link heavy reliance on web-based media to decreased white matter integrity, crucial for language skills. Adolescents may be more vulnerable to anxiety and unrealistic expectations due to digital media overuse.

Digital media overuse impacts brain development, especially cognitive and inhibitory control, attention, memory, and reasoning, essential for adapting to dynamic environments. Early exposure to fast-paced media can impair motor skills, spatial awareness, problem-solving, and language learning. Neuroimaging studies reveal that environmental factors like screen usage affect brain networks controlling social-emotional behavior and executive functions. Overreliance on smartphones diminishes gray matter in key brain regions, affecting cognitive and emotional regulation.

The internet generation, characterized by advancements such as Web 3.0, introduces artificial intelligence and semantic web technologies, reshaping digital content processing. The neurobiological basis of digital dementia involves changes in the brain structure and function, with excessive screen exposure linked to cognitive impairments. Neuroplasticity, or the brain's adaptability, plays a role in cognitive decline from digital media overuse. Early childhood and adolescent brain development stages exhibit significant plasticity, influencing cognitive trajectories.

Addressing digital dementia requires strategies to reduce screen time, promote cognitive exercises, and enhance awareness. Parents should regulate children's screen usage, encourage digital detox periods, and substitute screen time with other activities. Cognitive training programs such as Cogmed (Neural Assembly Int AB, Stockholm, SWE) and CogniFit (San Francisco, CA, USA) can improve memory and attention in older adults. Promoting balanced technology use and educating on the risks of excessive digital media consumption is crucial for maintaining cognitive health in the digital age.

## Introduction and background

Dementia is a group of symptoms associated with a decline in cognitive function, which may arise from various conditions that progressively damage brain tissue by harming cholinergic and glutamatergic neurons. This damage ultimately impairs cognitive abilities, such as memory, reasoning, and problem-solving, beyond what would be expected from the normal aging process. While consciousness remains unaffected, state of mind, regulation of emotions, behavior, and inspiration are frequently altered along with, and sometimes before, the decrease in cognitive ability [1]. In 2012, German neuropsychiatrist Dr. Manfred Spitzer proposed the idea of 'digital dementia,' which emphasizes the possible harm to the brain that might result from an over-reliance on technologies [2] such as cellphones and Google that can exacerbate attention deficit hyperactivity disorder (ADHD), memory loss, and cognitive decline. Since the release of Dr. Spitzer's *Digital Dementia*, a book focusing on the effects of gaming on children, the phrase has become more widely used. The phrase originated from news articles concerning memory issues among overworked Koreans. As a result of its many translations, the book acquired alarming and popular titles such as *Digital Dementia: How We Deprive Ourselves and Our Children of Reason* [3]. The widespread incidence of digital dementia is starkly illustrated by research, especially when it comes to the younger demographic. In recent WHO research, more than 90% of teenagers globally reported utilizing electrical appliances for at least two hours each day, with a considerable percentage going above and beyond two hours [4]. There is an urgent need for interventions because rates of cognitive impairment and attention deficiencies have dramatically escalated in combination with increasing dependency on screens [5].

There has been an observable increment in symptoms related to digital dementia in countries with high penetration of smartphone and internet usage, including South Korea, Japan, the U.S., and several European nations. In a South Korean study, one of the most digitally interconnected countries, around 18.4% of adolescents were reported to be at high risk for smartphone addiction. Notable correlations exist between heavy use of digital gadgets and cognitive impairment symptoms such as memory problems and attention deficits. In South Korea and Japan, where over 90% of smartphone penetration among youth is common, studies indicate that excessive usage of gizmos is associated with reduced levels of concentration as well as poor memory in students. Similarly, digital overuse remains a source of worry within the U.S. and Europe. An investigation shows that young adults from the U.S. who indulge too much in mobile phone usage usually experience more signs associated with attention deficit hyperactivity disorder (ADHD), which is similar to symptoms indicating digital dementia [3,5-7].

The fifth generation (5G) of the internet has enabled populations to have fast internet service across the country. The primary issues with internet generation include speed, capacity, coverage, rates of data, requirements, and new features. Every 10 years, users require a new generation to prevent these issues [6]. The internet generation is classified according to the following factors: system architecture, language, technology, timeline, processing mode, and features. Digital technology refers to the use of desktops, the internet, mobile apps, and mobile phones and tablets such as smartphones in the psychological treatment of mental health conditions. It is changing clinical practice, and services, and aiding the spread of treatment globally, impacting a range of uses and applications [5].

Digital media use has been found to affect motor skills, language, cognition, and perception of visual objects in the developing brain [6]. In empathy studies in young adults, a correlation between time spent with digital media and a lower cognitive empathy with other humans has been reported. This may be due to a lack of insight into what other people might think (theory of mind), problems with facial recognition, or lack of exposure to peers (due to excessive time spent online) [8]. Some studies report no correlation between online time and empathy. The advancement of language-related processes (vocabulary and interpretation) is also impacted by heavy reliance on web-based media. As a whole, it has been shown that there is a direct link between extensive use of electronic media in early infancy and decreased white matter tract microstructural authenticity, particularly between the Wernicke and Broca regions of the brain [9,10]. The emergence of these fiber tracts is strongly connected with understanding languages and abilities. To determine the ramifications for the growing brain, especially during the first years of life and the phases of evolving neurological development, more research is required. Reading skills might be compromised if fiber tracts between language areas are not developed to their full extent. Due to psychological predisposition, network compliance, and social awareness, teenagers may be more susceptible to fabricated headlines, unreasonable expectations, and anxiety disorders. This is especially the case when it comes to the detrimental usage of digital media [11].

This review looks at the way digital technologies affect mental processes like memory, attention, and judgment. It draws attention to the variations in impacts among various generations and discusses ways to optimize advantages while minimizing disadvantages. It highlights the necessity of more investigation and well-informed decision-making for the best possible cognitive function and well-being in the digital age.

## Review

Selection of Studies

The selection process for the studies involved several stages. Initially, two reviewers independently screened the titles and abstracts of all identified studies. Studies were included if they focused on the cognitive impacts of digital technology use, including memory, attention, and overall cognitive decline. Any discrepancies between the reviewers were resolved through discussion and consensus. This stage resulted in the exclusion of 923 articles, leaving 434 for full-text review. Next, the full texts of the remaining 434 articles were assessed for eligibility based on specific inclusion criteria. These criteria included studies examining the concept and symptoms of digital dementia, research on the cognitive impact of digital technology use across different age groups (children, adolescents, young adults, and older adults), articles discussing the neurobiological basis and mechanisms of digital dementia, and studies proposing management strategies for digital dementia, including screen time reduction and cognitive exercises. Studies were excluded if they did not directly address cognitive function in the context of digital technology use, and if they were not empirical studies (e.g., opinion pieces, editorials) or were duplicates. This thorough review process led to the exclusion of 332 studies.

The remaining 102 articles were then subjected to data extraction and quality assessment. Data on study design, sample size, population characteristics, interventions, outcomes, and key findings were extracted. The quality of each study was assessed using standardized criteria, focusing on aspects such as study design, methodological rigor, and relevance to the research questions. Studies that did not meet the quality criteria were excluded, resulting in 78 high-quality studies in the final review. The final selection of 78 studies provided a comprehensive overview of the current state of research on digital dementia and the cognitive impacts of digital technology use. These studies were synthesized to identify common themes, gaps in the literature, and potential areas for future research. The findings inform the discussion and recommendations presented in this review.

Understanding digital dementia

Concept and Origin of Digital Dementia

Digital dementia, first introduced by Spitzer in 2012, is a condition in which individuals' memory and calculation abilities decline due to excessive reliance on digital devices. This decline results from unconscious reliance on digital devices, leading to symptoms of forgetfulness. According to Arakelyan, digital dementia is a condition that impairs immediate recall, with synaptic pathways deteriorating due to excessive use of technology [12]. New behaviors such as using Google to search for information may increase digital dementia. The term can be interchangeable with terms like 'digital amnesia' and 'the Google Effect' [13]. Additionally, while excessive dependence on digital communication can reduce face-to-face social interactions and potentially foster social isolation, which may lead to changes in brain structure and function, particularly in areas related to social cognition and emotional regulation, there is evidence that moderate and mindful use of digital media can have neutral or even positive effects on cognitive function and social well-being, highlighting the need for more objective data on optimal usage levels [14].

Symptoms and Cognitive Impact

The pathophysiological mechanism underlying digital dementia involves a multifaceted sequence of adverse effects precipitated by excessive screen time. Initially, prolonged engagement with digital devices, such as smartphones, tablets, and computers, contributes to a sedentary lifestyle characterized by markedly reduced physical activity. This inactivity impedes the neuroplasticity of the brain, as physical exercise is known to enhance synaptic connectivity and overall neural health [14,15].

Concurrently, the cognitive burden imposed by constant multitasking and rapid switching between tasks leads to cognitive overload. This state of cognitive strain results from the brain’s effort to process an excessive volume of information from continuous notifications and digital media consumption. Such overload can detrimentally affect executive functions, including working memory and attention [16].

Furthermore, exposure to blue light from screens for an accumulation of more than eight hours a day disrupts the circadian rhythm, thereby impairing sleep quality and reducing sleep duration. Sleep disturbances are well-documented to exacerbate cognitive decline, as they hinder the brain’s ability to consolidate memories and perform essential restorative functions [17]. Additionally, the reduction in face-to-face social interactions due to increased dependence on digital communication fosters social isolation. This isolation can lead to alterations in brain structure and function, particularly in areas associated with social cognition and emotional regulation [14].

**Figure 1:**
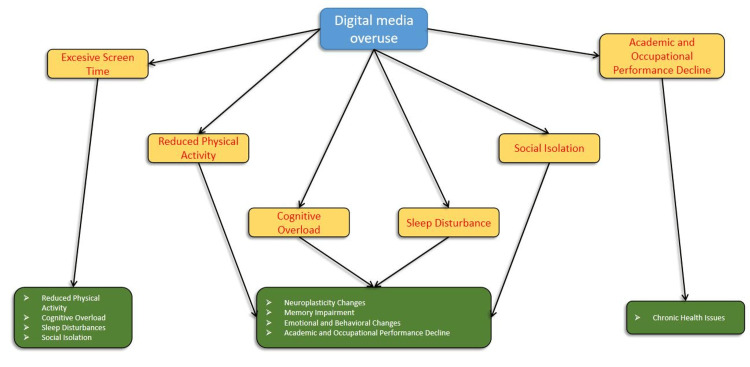
Pathophysiological mechanism of digital dementia Yellow boxes: Mechanisms; Green boxes: Outcomes Diagram created by the authors.

Collectively, these factors contribute to significant neuroplasticity changes, including decreased synaptic plasticity and impaired neural connectivity [12]. The resultant cognitive impairments manifest as deficits in both short-term and long-term memory, as well as difficulties in concentration and information recall. Emotional and behavioral changes, such as increased stress, anxiety, mood swings, and depressive symptoms, further compound the impact on cognitive health. Consequently, individuals may experience a decline in academic and occupational performance, characterized by decreased productivity and an increased propensity for errors. Over time, these issues can culminate in chronic health conditions, such as obesity and cardiovascular diseases, underscoring the extensive and multifactorial impact of digital dementia [13-15]. This mechanism has been elaborated in Figure 1.

Memory Impairment

Screen time for more than two hours a day (as mentioned in the National Institutes of Health study) has a detrimental effect on brain growth and structure, especially on cognitive and inhibitory control, concentrated attention, memory, intellectual reasoning, and artistic ability [12,13]. These fundamental abilities underpin human intellect and the capacity to adjust to unpredictable, dynamic, and open-ended settings. They are also necessary for managerial leadership in goal-directed and decision-making activities. These cognitive-behavioral talents may continue into early and midlife and increase susceptibility to expedited neurodegeneration in late adulthood if the brain circuits underpinning them are underdeveloped or abnormally formed prior to maturity [13]. Overexposure (more than seven hours a day) to screens has been associated with developmental problems in the areas of motor skills, spatial awareness, problem-solving, and language learning. Deficits in cognition and behavior may result from early childhood exposure to fast-paced television [14]. A crucial component of the reward circuit pathway that leads to substance abuse problems in teenagers and young adults is the orbitofrontal thickness, which is considerably diminished in children exposed to two hours or more of screen time each day and in those exposed to seven hours or more. Post-secondary students who were not allowed to use electronic devices in class performed better on exams, demonstrating the long-term consequences of digital technology on maturity. It has been reported that the lasting effect may be for years. Decreases in long-term memory and cognitive growth are among the long-term impacts. Many adults no longer practice self-imposed analytical thinking; instead, they rely heavily on technology and smartphones [16,17].

Attention Deficits

The prenatal and adolescent transitions, in particular, are times of extreme flexibility during the phases of brain development. According to neuroimaging research, several dynamic brain networks that control intellect, social-emotional behavior, and executive processes form in early brain development and become more functionally interconnected during adolescence [18]. Different patterns of connection can be used to forecast both sick and healthy adult development paths. Early environmental events, such as childhood abuse, living in an urban setting, and screen usage, are thought to have an impact on changes in these cortical networks [18]. Attentional issues are linked to early television exposure; 10% of children aged one to three experience attentional issues as early as age seven. Teenagers and young adults are more likely to have poor academic development if they multitask on digital devices more often, especially when they are studying. For people with the greatest degree of smartphone dependability, the usage of devices can have a deleterious impact on the capacity for working memory and operational cognitive abilities [18,19].

Reduced Cognitive Processing Abilities

Overusing screens can harm cognitive functioning, such as memory, intellectual thinking, imagination, and cognitive and inhibitory control. The executive functions of these cognitive abilities include directed behavior towards a goal, making decisions, and the ability to adapt to novel and ever-changing environments [20]. The prefrontal cortex, responsible for higher cognitive functions, is especially sensitive at this period of growth. Long-term screen exposure can disrupt neurons connected to cognitive-behavioral processes in an abnormal manner. For example, research shows that incessant interaction with screens may interfere with normal neural circuit development related to concentration, liquidity of memories, and flexibility of thought processes. This impairment affects executive function, which plays an important role in problem-solving skills and decision-making [21].

The internet generation

Characteristics of the Internet Generation

A recent advancement in the internet, known as Web 3.0, blends artificial intelligence, semantic web technology, and decentralization. Through a peer-to-peer network, it seeks to transfer power from entities in charge of data to end users. By adding machine-interpretable metadata to the web, the semantic web seeks to improve its usability and enable computers to comprehend and interpret data. Digital content will be interpreted by artificial intelligence, making it possible for robots to comprehend and translate information at a level comparable to that of humans. Semantic and natural language processing skills work together to give computers the ability to process information more quickly and accurately [19-21].

Digital Natives vs. Digital Immigrants

'Digital immigrants' are people who learn how to use a computer when they are older, whereas 'digital natives' are those who are younger and are born into the digital age. Whereas digital natives are usually expected to be digitally literate from birth, immigrants are frequently believed to have some difficulty with technology-related matters. Instead of seeing digital natives and immigrants as either/or, the authors contend that there is a continuum that is best described as digital fluency. They put out an exploratory conceptual model of digital fluency that lists variables that affect digital fluency both directly and indirectly [22].

Patterns of Technology Use

Depending on one's physical capabilities and level of handicap, technology utilization varies. Comparable rates of internet and e-mail/text messaging use are seen among those with less severe disabilities and more physical capabilities for interaction. However, the usage of electronic devices is far lower among people with more severe disabilities, such as those who need assistance with ADLs or movement beyond the house. The usage of technology differs according to symptoms and disabilities that restrict activities. Compared to older persons without visual or memory difficulties, those with these conditions are less likely to utilize technology. Lack of funding, restrictions on web page design, and rules that do not specifically encourage accessible design are some of the obstacles to the use of technology. Further study is required to determine how different types of impairments utilize technology differently and whether performance expectations influence a person's decision to use technology [23,24].

Impact of Technology on Daily Life and Cognitive Habits

Individual minds and personalities have been profoundly affected by technological advances, both favorably and unfavorably. The generation of structural and functional changes brought on by digital gadgets depends heavily on the brain's capacity for change adaptation. Regular screen time alters cortical activity, which causes the motor and sensory cortex to grow. In the developing brain, neuroplasticity processes are very active, particularly during the dynamic brain development that occurs in early life. Adolescence is a period of profound brain growth, characterized by noticeable alterations in the brain regions responsible for emotional and social behavior. Evidence for both acute and persistent abnormalities in particular cognitive domains is provided by the neurobiological processes behind internet and gaming disorder (IGD), which may represent structural and functional changes in the brain [25].

Multitasking and Attention Spans

People can now routinely participate in numerous mediated tasks at once (multitasking) because of advancements in media technology. Multitasking with media can also be used to divert attention from low-stimulation or boring chores. This study looked into how people's assessments of advertising are impacted by multitasking. While the advertisements were running, participants could choose to complete additional tasks or just watch the advertising. The findings demonstrated that when participants completed extra on-screen activities while ads were airing, they felt as though time was going more rapidly than when they merely watched the commercials. Additionally, multi-window multitasking enhanced the satisfaction of the activity overall and raised the assessments of the advertisements; this effect was mediated by the impression of time passing swiftly while the advertisements were playing [26].

Dependency on Digital Devices for Information

Personal digital dependency exists beyond smart devices serving as personal assistants. The copious amounts of information generated by mobile applications can be detrimental as well as helpful, particularly for young users. Unwanted and deceptive material is shared on social media platforms such as Facebook, Instagram, Telegram, WhatsApp, and Twitter, which increases reliance on smart gadgets and alters behavior. The reliance on these gadgets has grown significantly due to this knowledge [27,28].

Mechanisms of digital dementia on a neurological basis

Changes in Brain Structure and Function

Overexposure to screens has been linked to difficulties reaching developmental milestones for language acquisition, problem solving, motor skills, and spatiotemporal abilities. For both newborns and children, attention, learning, and memory impairment were linked to exposure level, content, and tempo [29]. Wakeup prompts individuals to use their phones to browse social media or apps, a mindless, automated process that rewards them with dopamine. This lack of self-regulation can make these chemicals addictive, lazy, and negatively impact an individual's emotional state [30]. Multifunctional neural connections that control intellect, social-emotional behavior, and executive processes arise early during childhood and become more pronounced throughout teenage years, according to neuroimaging research. These networks have the ability to forecast both normal and abnormal growth in adolescents. Environmental influences, such as excessive screen time, can influence these networks. If these neural circuits are underdeveloped before adulthood, they may persist into early and middle adulthood, increasing the risk of early-onset mild cognitive impairment (MCI) and Alzheimer’s disease and related dementias (ADRD). Overuse of social media can reduce the amount of gray matter in the lingual gyrus, insula, anterior and posterior cingulate cortices, and amygdala [16].

Neuroplasticity and Its Role in Cognitive Decline

The term 'neuronal plasticity' describes the ways in which experience alters neurons, such as via neurogenesis, synaptogenesis, dendritic arborization, and network rearrangement. The effects of digital media on cognitive functions are mixed, with both beneficial and detrimental effects depending on usage. Excessive touchscreen use on smartphones can reshape the somatosensory cortex, while excessive internet use can negatively affect memory and attention. Video games can refine orientation and spatial abilities, but uncontrolled use can lead to distraction and reduced sustained attention. Social media can cause attentional overload and affect academic performance. Brain imaging reveals lower white matter tract integrity and structural impairments in brain regions [31]. 

Comparing digital dementia across age groups

Impact on Children and Adolescents

Digital use in children can increase cognitive skills, creativity, and digital literacy. However, excessive use can lead to negative outcomes like decreased attention intervals, lower academic performance, and socio-emotional challenges. Mobile device use in toddlers can also lead to lower expressive language skills and cognitive development deficits. Therefore, it's crucial to balance digital use with other aspects of childhood [32]. The term 'experience-dependent plasticity' refers to the capacity of the brain to change its structure and function as experiences and environmental stimuli. This neuroplasticity is critical for learning and adaptation over the life span, but it is particularly pronounced during early developmental stages. On the other hand, cognitive load theory explains how the brain processes material through control of intrinsic, extraneous, and germane cognitive loads. In this regard, there is intrinsic load, which has to do with the intrinsic complexity of the information itself; extraneous load entails nonessential information that can exceed cognitive ability; while germane load is related to effort made in forming a long-term memory trace [32,33].

Effects on Young Adults

Digital technology usage among young adults is highly prevalent, with social networking, social media, and online communication channels being the most common. Their socioemotional state, academic achievement, and mental health may all be adversely affected by this consumption. Using digital communication sites, like Facebook, has been on a regular basis (once per day, for a minimum of an hour) connected to worse academic achievement, bad psychological consequences, and cultural pressures. The effects of internet use on young adults can be mitigated, though, by developmental variables including learning time management techniques and striking a balance between online and offline activities. Teenage depression symptoms and feelings of isolation have also been connected to frequent social media use. Vulnerability to peer pressure and a lack of self-regulation abilities may make these effects more pronounced [33].

Cognitive Decline in Older Adults With High Technology Use

The use of electronic media with senior citizens is growing due to its potential advantages. These include improved cognitive performance, more social interaction, and easier access to health data. Memory, attention, and executive function may all be enhanced by cognitive training using digital platforms, such as programs for computers, smartphone applications, wearable technology, or web-based tools. The use of online platforms has been linked to increased social support and decreased feelings of loneliness. Using digital technology can help with memory, focus, and resolving problems in specific brain-training applications, and exciting games can do this. The beneficial effects of digital technology may depend on a variety of factors, including personal preferences, prior experience, and digital proficiency. There may be difficulties, including social isolation, cognitive deterioration, and usability limitations [34]. For each age group, age-appropriate guidelines and interventions are required to address the advantages and disadvantages of digital technology. Table 1 shows the studies reviewed for mechanisms of digital dementia and their findings or solutions for the same.

**Table 1:** Studies reviewed for explaination of digital dementia and the corresponding findings

Study	Mechanism of digital dementia	Findings/suggestions
Madigan et al., (2019) [29]	Neurobiological basis: Changes in brain structure and function	Overexposure to screens linked to developmental difficulties in language acquisition, problem-solving, motor skills, and spatiotemporal abilities. Impairments in attention, learning, and memory correlated with exposure level, content, and tempo.
Horoszkiewicz, (2022) [30]	Neurobiological basis: Changes in brain structure and function	Wakeup prompts for phone usage to browse social media or apps create a mindless, automated process rewarding dopamine, leading to addiction and negative emotional impacts.
Barros, (2024) [31]	Neuroplasticity and its role in cognitive decline	Digital media can alter neurogenesis, synaptogenesis, dendritic arborization, and network rearrangement. Excessive touchscreen and internet use affect memory and attention, while video games can improve spatial abilities but also reduce sustained attention. Brain imaging shows lower white matter tract integrity and structural impairments in brain regions.
Radesky et al., (2015) [32]	Comparing digital dementia across age groups: Impact on children and adolescents	Digital use can increase cognitive skills, creativity, and digital literacy, but excessive use leads to decreased attention, lower academic performance, and socio-emotional challenges. Mobile device use in toddlers can cause cognitive development deficits.
Odgers & Jensen, (2020) [34]	Comparing digital dementia across age groups: Effects on young adults	High digital technology usage in young adults can negatively impact socio-emotional state, academic achievement, and mental health. Frequent social media use is linked to depression and feelings of isolation. Developmental factors like time management skills can mitigate effects.
Ziegler et al., (2022) [33]	Comparing digital dementia across age groups: Cognitive decline in older adults with high technology use	Digital technology can enhance cognitive performance, social interaction, and health data access in older adults. Cognitive training through digital platforms can improve memory, attention, and executive function. However, overuse can lead to social isolation, cognitive decline, and usability issues.
Cerniglia et al., (2017) [28]	Neurobiological basis: Changes in brain structure and function	Excessive screen time influences multifunctional neural connections controlling intellect, social-emotional behavior, and executive processes, potentially leading to early onset mild cognitive impairment (MCI) and Alzheimer's disease and related dementias (ADRD).
Panjeti-Madan & Ranganathan, (2023) [35]	Comparing digital dementia across age groups: Impact on children and adolescents	Screen time impacts children's development in cognitive, language, physical, and socio-emotional domains. Excessive use can lead to various developmental issues.
Eckroth-Bucher & Siberski, (2009) [36]	Neuroplasticity and its role in cognitive decline	Integrated cognitive stimulation and training programs can preserve cognition. Digital platforms need to be balanced to prevent cognitive decline and maintain cognitive health.
Irazoki et al., (2020) [37]	Comparing digital dementia across age groups: Cognitive decline in older adults with high technology use	Cognitive training and rehabilitation through technology for people with mild cognitive impairment and dementia show promising results in maintaining and enhancing cognitive functions.

Table 2 summarizes studies on digital dementia, highlighting the primary mechanisms and the number of studies for each. It shows that changes in brain structure and function are the most studied mechanism (four studies), followed by neuroplasticity (two studies), and age group comparisons (six studies in total).

**Table 2:** Summary of studies reviewed for explaination of digital dementia

Mechanism of digital dementia	Number of studies	Studies reviewed
Neurobiological basis: Changes in brain structure and function	4	Madigan et al., 2019 [29]; Horoszkiewicz, 2022 [30]; Cerniglia et al., 2017 [28]; Barros, 2024 [31]
Neuroplasticity and its role in cognitive decline	2	Barros, 2024 [31]; Eckroth-Bucher & Siberski, 2009 [36]
Comparing digital dementia across age groups: Impact on children and adolescents	3	Radesky et al., 2015 [32]; Panjeti-Madan & Ranganathan, 2023 [35]; Odgers & Jensen, 2020 [34]
Comparing digital dementia across age groups: Effects on young adults	1	Odgers & Jensen, 2020 [34]
Comparing digital dementia across age groups: Cognitive decline in older adults with high technology use	2	Ziegler et al., 2022 [33]; Irazoki et al., 2020 [37]

Management of digital dementia

Strategies for Reducing Screen Time

Parental controls on children's screen usage should be implemented. These precautions include choosing media content carefully, implementing co-viewing, imparting viewing skills, limiting the amount of time spent watching media, refraining from using media as a calming tool, promoting digital detox time, removing electronic devices from children's bedrooms, adhering to the 20-20-20 rule, lowering their own screen-time limits, substituting screen time with other activities such as outdoor games, learning skills, sleep, social interaction, studies, and exercise, and being informed about the potential negative effects of excessive media and screen usage. By taking these precautions, parents may help their children learn healthy viewing habits and guard against digital visual strain. Furthermore, parents ought to exercise caution when substituting screen time for other activities in the development of their children [35]. Restrict device updates, spend a shorter period watching entertainment passively, find other things to concentrate on, and schedule downtime for rest and connection to lessen your reliance on technology. Using applications to restrict scrolling time or combining screen-time activities like reading a book and watching TV are two ways to reduce screen time. Establishing a timetable for these pursuits can aid in preserving equilibrium and lessening the detrimental effects of technology in one's daily activities [10].

Cognitive exercises and activities

Memory Training

Captain's Log (BrainTrain Inc., North Chesterfield, VA, USA) is a cognitive training program used in an integrated cognitive stimulation and training program. A study with participants with mild and moderate impairment and healthy older adults showed that a combination of stimulation techniques improved logical memory domain eight weeks post-intervention [36]. Cogmed (Neural Assembly Int AB, Stockholm, SWE), CogniFit (San Francisco, CA, USA), and CogniPlus (Schufried, Mödling, AUT) are computerized cognitive training exercises whose efficacy has been assessed in three separate trials. In older persons with MCI, Cogmed demonstrated improvements in perceived issues with memory and behavioral working memory. Compared to participants with MCI, those with mood-related neuropsychiatric symptoms (MrNPS) performed better on CogniFit in terms of global cognitive capacity. CogniPlus outperformed conventional group-based programs in cognition, attention, and quality of life. These studies demonstrate how computational mental exercise can enhance cognitive function and overall quality of life [37].

Attention Enhancement Techniques

For both physical and emotional well-being, regular physical activity is essential. Examples of this include yoga, walking, and fitness courses. It gives you more energy, lowers tension, and keeps you away from electronics. Without relying on technology, enjoying fun activities such as sports, cycling, or dancing may improve mood and produce endorphins, which lower stress and increase energy.

Role of education and awareness

Promoting Balanced Technology Use

A 'digital detox' is undertaken to enhance tech-life balance and lessen one's reliance on technology. This purposeful abstention may prevent cognitive deterioration and enhance cognitive functioning. Depending on the demands of the individual, the advantages of digital detoxification may take many forms, such as restricting one app or gadget or giving up technology for seven days. It can also involve being device-free once a week. Studies indicate a connection between a healthy diet and improved sleep quality. A healthy, well-balanced diet can improve general well-being and lessen the need for excessive screen time. Fruits and vegetables, lean protein, healthy fats, complex carbs, foods high in calcium, magnesium, tryptophan, and water are among the recommended nutrients. In addition to supporting mental health, these meals lower inflammation, control blood sugar, encourage calmness, and keep children hydrated. Better sleep and general health can be enhanced by encouraging children to consume a range of fruits and vegetables, lean protein, healthy fats, and complex carbs [34].

## Conclusions

The mental health of children and teenagers, their social connectivity, and behavioral traits such as narcissism and empathy have been raised by the issue concerning a digital lifestyle. Encouraging media use that positively affects joy, fulfillment in life, and positive social attitudes in youths requires an understanding of these effects and research gaps. Results on well-being and intricate relationships between individual factors impact social connectivity, empathetic thinking, personality, variables, digital media interaction type, and media-contextual experiences. Further investigation is required to determine the ways, locations, timing, and individuals for whom digital media activities promote outcomes related to social connectivity and good mental health. It is also necessary to investigate media consumption trends over time and their associated effects and conduct longitudinal studies. Certain technologies can be created to promote prosocial personality traits, social results, and good well-being.

Adolescents are particularly vulnerable to the negative effects of high digital exposure due to their developing brains, which are highly plastic and sensitive to external stimuli. For young adults, frequent use of digital platforms, especially social media, has been linked to adverse outcomes, including diminished academic performance and mental health issues. In older adults, while digital technology can offer cognitive benefits such as enhanced memory and attention through targeted applications, overuse can contribute to cognitive decline and social isolation. Strategies to mitigate the risks of digital dementia include the reduction of screen time, promotion of alternative cognitive exercises, and enhancement of public awareness and education about healthy digital habits. Further research is needed to determine optimal usage levels and to develop tailored interventions for different age groups to maximize the cognitive benefits of digital technology while minimizing its potential harms.
